# Nursing and midwife staffing needs in maternity wards in Burkina Faso referral hospitals

**DOI:** 10.1186/1478-4491-12-S1-S8

**Published:** 2014-05-12

**Authors:** Antarou Ly, Séni Kouanda, Valéry Ridde

**Affiliations:** 1 Département de santé publique, Institut de recherche en Sciences de la santé (IRSS), 03 BP 7192 Ouagadougou, Burkina Faso; 2 Kaya health and Demographic surveillance system, Kaya HDSS, Kaya, Burkina Faso; 3 Institut Africain de santé publique, 12 BP 50 Ouagadougou, Burkina Faso; 4 Centre de recherche du Centre hospitalier de l’Université de Montréal (CRCHUM), Montreal, Canada; 5 Department of Social and Preventive Medicine, University of Montreal, Montreal, Canada

**Keywords:** workload, health worker, hospital referral, charge de travail, travailleur de la santé, hôpital de référence

## Abstract

**Background:**

In 2006, Burkina Faso set up a policy to subsidize the cost of obstetric and neonatal emergency care. This policy has undoubtedly increased attendance at all levels of the health pyramid. The aim of this study was to measure the capacity of referral hospitals’ maternity services to cope with the demand for health services after the implementation of this policy.

**Methods:**

This study was conducted in three referral health centres (CMAs, CHRs, and CHUs). The CHU Yalgado Ouédraogo (tertiary level) and the CMA in Sector 30 (primary level) were selected as health facilities in the capital, along with the Kaya CHR (secondary level). At each health facility, the study included official maternity ward staff only. We combined the two occupational categories (nurses and midwives) because they perform the same activities in these health facilities. We used the WISN method recommended by WHO to assess the availability of nurses and midwives.

**Results:**

Nurses and midwives represented 38% of staff at the University Hospital, 65% in the CHR and 80% in the CMA. The number of nurses and midwives needed for carrying out the activities in the maternity ward in the University Hospital and the CMA is greater than the current workforce, with WISN ratio of 0.68 and 0.79 respectively. In the CHR, the current workforce is greater than the number required (WISN ratio = 2).

This medical centre is known for performing a high number curative and preventive activities compared to the Kaya CHR. Like the CHU, the delivery room is a very busy unit. This activity requires more time and more staff compared to other activities.

**Conclusion:**

This study showed a shortage of nurses and midwives in two health facilities in Ouagadougou, which confirms that there is considerable demand. At the Kaya CHR, there is currently enough staff to handle the workload in the maternity ward, which may indicate a need to expand the analysis to other health facilities to determine whether a redistribution of health human resources is warranted.

## Abstract (fr)

**Contexte:**

En 2006, le Burkina Faso a instauré une politique de subvention des coûts des services d’urgences en obstétrique et en soins néonataux. Cette politique a indéniablement augmenté l’achalandage à tous les niveaux de la pyramide de la santé. L’objectif de la présente étude était de mesurer la capacité des services de maternité des hôpitaux de référence à absorber la demande accrue de soins de santé à la suite de la mise en oeuvre de cette politique.

**Méthodes:**

L’étude a été réalisée dans trois centres hospitaliers de référence (CMA, CHR et CHU). Nous avions choisi le centre hospitalier universitaire Yalgado Ouédraogo (3e niveau) et le Centre médical avec antenne chirurgicale du secteur 30 (1er niveau), comme établissements de santé dans la capitale, et le Centre hospitalier régional de Kaya (2e niveau). Dans chaque établissement de santé, l’étude n’a porté que sur le personnel officiel des services de maternité. Nous avons combiné les deux catégories professionnelles (personnel infirmier et sages-femmes), car elles effectuent les mêmes activités au niveau de ces formations sanitaires. Nous avons utilisé la méthode WISN (indicateurs de la répartition du travail pour la dotation en personnel) conçue par l’OMS pour évaluer la disponibilité du personnel infirmier et des sages-femmes.

Trois centres de santé à trois niveaux de la pyramide de la santé du Burkina Faso ont été inclus dans l’étude.

**Résultats:**

Le personnel infirmier et les sages-femmes représentaient 38 % du personnel du CHU, 65 % du CHR et 80 % du CMA. Le nombre d’infirmières et de sages-femmes dont on a besoin pour effectuer les activités du département de maternité au CHU et au CMA était plus élevé que la main-d’oeuvre existante selon le ratio WISN de 0,68 et 0,79, respectivement. Dans le CHR, la main-d’oeuvre existante était plus grande que le nombre requis (ratio WISN= 2).

Ce centre médical est caractérisé par une forte affluence des activités curatives et préventives par rapport au CHR de Kaya. Tout comme le CHU, la salle d’accouchement est une unité à forte activité. Cette activité nécessite plus de temps et plus de personnel comparativement aux autres activités.

**Conclusion:**

Cette étude a montré un manque en personnel infirmier et de sages-femmes au niveau de deux établissements de santé de la capitale, Ouagadougou, confirmant la forte pression de la demande. Au CHR de Kaya, l’effectif est pour l’instant suffisant pour faire face à la charge de travail en maternité, ce qui indique le besoin d’étendre l’analyse à d’autres établissements de santé afin de déterminer si une redistribution des ressources humaines est justifiée.

## Introduction

One of the challenges facing developing countries, particularly in Africa, in achieving the Millennium Development Goals (MDGs) is the availability of health human resources. Not only do these countries have a shortage of human resources, but their distribution is often uneven, especially between urban and rural areas [1,2]. In light of this shortage of health human resources, African countries have implemented policies to retain staff in rural areas. In parallel with such a retention policy, several countries have also implemented policies (e.g. free-access or subsidies) to relieve the population of the financial burden associated with accessing care [3,4]. This policy has resulted in an increased use of health services, particularly by women and children under five years of age. However, studies have shown that, in Ghana and South Africa, these policies are accompanied by a shortage of health workers and by adverse effects on their practices [5,6].

Given the scarcity of health human resources and the increased use of health services, health workers often complain of being overworked. Burkina Faso, a French-speaking West African country, is also affected by this health human resources issue. In 2003, the State adopted a policy for the regionalization of health worker recruitment in order to promote better geographical distribution of health workers [7]. Even before this policy, we noticed a poor geographic distribution of health workers in the country, focused on the two major cities, Ouagadougou and Bobo Dioulasso [8]. Following implementation of the childbirth and emergency obstetric and newborn care (EmONC) subsidy policy in 2006, an increase in the use of health services [9] by women was noted in several health districts in Burkina Faso from 2006 to 2009. In turn, this led to an increase in primary health centre (CSPS) referrals toward higher levels of care (Medical Centre with Surgical Unit (CMA), regional hospital (CHR) and National University Teaching hospital (CHU). From 2003 to 2009, the rate of assisted delivery was doubled from 30.92 % to 73.40 % nationally [9]. In one district of the country, the rate rose from 49% in 2006 to 84 % in 2010 [10]. This subsidy covers 80% for normal deliveries at the first level of care, that is, in CSPSs and CMAs; 60% for deliveries at the second and third levels of care, namely, CHRs and CHUs; and 80% for emergency deliveries in CMAs, CHRs, and CHUs [9].

We conducted a study on workload in first-line health facilities in Burkina Faso (CSPSs). The results showed that health centres have enough health workers to manage the workload of a free health care system [11].

However, the empirical data indicates that health care workers, particularly at the referral levels, complain of being overworked. Similar studies have been conducted in a number of English-speaking African countries [12-16]. There is currently a lack of data on French-speaking West African countries.

The purpose of this article is therefore to examine the ability of referral hospital maternity wards to meet the public's demand for services following EmONC intervention.

This study focuses primarily on nurses and midwives, which are the occupational categories that attend to the majority of maternity activities (i.e. antenatal visits and deliveries).

## Methodology

This study was conducted in referral health centres (CMAs, CHRs, and CHUs) which represent the three levels of the health pyramid of Burkina Faso. We had selected the CHU Yalgado Ouédraogo (tertiary level) and the CMA in Sector 30 (primary level) as health facilities in the capital (urban areas), and the Kaya CHR (secondary level) in a rural area. We chose these two centers in the capital city because the health information system data showed a very high utilization of maternity services at the CHU (8752 inpatients and 3752 deliveries in 2011) and the sector 30 CMA (4199 inpatients and 3371 deliveries). The Kaya CHR was selected because of its health and demographic surveillance system (HDSS) that allowed us to conduct this study (2511 hospitalized patients and 1418 deliveries in 2011).

At each health facility, the study included official maternity ward staff only, to detect the maternity wards' ability to keep up in light of the EmONC policy. We combined the two occupational categories (nurses and midwives) because they perform the same activities in these health facilities.

We used two tools to collect data: (i) an observation guide to observe and time various tasks performed by midwives and nurses; and (ii) a retrospective activities census guide completed in 2011. In total, the data collection lasted 34 days: 14 days at the CHU, 10 days at the CHR, and 10 days at the CMA. Six investigators were recruited and trained to collect data in the field.

We used the Workload Indicators of Staffing Need (WISN) method to calculate nursing and midwife staffing needs in these maternity wards. This method was developed by the World Health Organization (WHO) to calculate optimal distribution and staff deployment [17]. It is a human resources management tool that (i) determines how many staff are needed to handle the workload of a health care facility, and (ii) assesses the workload pressures of health workers in that facility using the WISN ratio (actual staff to required staff). It takes into account annual work volume statistics, work time available for health workers, and how long it takes them to complete an activity. This method enables a link between supply (presence of human resources) and demand (activities volume). Using this tool, supply and demand can be measured in each health facility. Similar approaches have been used in Tanzania and Kenya [18,14]. The number of staff required in each maternity ward was calculated as follows:

- The available work time is the number of working days in a health worker's year (number of weeks per year (52) multiplied by the number of working days in a week (5) minus the number of non-working days, e.g. annual leave, public holidays, sick leave, training, etc.). Since nurses and midwives are public servants, their work time was calculated based on the standard work schedule for a public servant. The standard is five working days per week, eight working hours per day, 14 public holidays per year, and 10 authorized days of leave.

- The annual work time of each activity is the time spent performing the activity in one year, i.e., the duration of the activity multiplied by the total number of times the activity is performed in the year. The activities that comprise the workload were determined using the observation guide.

- To determine nursing and midwife staffing needs (A) for service activities, the annual work time for each activity included in the workload (using 2011 annual statistics) was divided by the total available annual work time. This equals the number of nurses and midwives required for that activity. For support activities, that is, activities in the health care centre for which no statistical data is available, the staffing need calculation is (B) = 1/[1-(total % of time to complete activities/100)];

- The percentage of time to complete activities per day is calculated as follows: (observed duration/8) x 100, with "8" being the number of working hours per day.

- The total number of nurses and midwives required is obtained by multiplying the number of nurses and midwives required for service activities by the number required for support activities (C = A x B). For this calculation, it was assumed that there were no individual activities because they perform the same activities. We used EpiData to enter the data and SPSS Statistics 17.0 and Microsoft Excel for analyses. We used the test for median comparison to interpret the observed durations in the three facilities.

## Results

This study examined the maternity wards of three health facilities. Each of them is comprised of several health care units, including 11 at the CHU, 9 at the CHR, and 8 at the CMA. In total, 12 health care activities were monitored at the CHU, 10 at the CHR, and 10 at the CMA (see Table 1). The units mostly consisted of the following rooms: delivery, postpartum care, post-op, resuscitation, intensive care, operating, family planning, manual vacuum aspiration (MVA), pathological pregnancy, and outpatient. There was also a working ultrasound room in the CHU, mainly used by physicians.

**Table 1:** Midwife and nurse activities by facility and observed median duration in minutes

	Observed median duration in min			
Activity	CHU	CHR	CMA	P-value

Delivery	127	83	173	0.080
Caesarean	15	13	16	0.211
Family planning consultation	16	15	11	0.000
Patient medical visit	10	-	6	0.001
Patient care	21	37	13	0.801
Completing admin/patient records	15	18	15	0.099
Manual vacuum aspiration (MVA)	33	22	30	0.000
Family planning counselling	19	-	-	-
Pre-caesarean care	43	60	74	0.223
Cervical cancer screening	21	7	-	1.000
Training (staff)	60	60	60	1.000
Dressing	180	180	180	1.000

### Breakdown of workload for nurses and midwives

Various activities were observed and comprise the workload. These activities are regularly performed by the nursing and midwife staff in these hospitals (Table 1). The support activities had very similar work times in all three health care facilities. The observed times vary from a few minutes to a few hours. These activities are not performed in the same manner at all health facilities. We note a significant difference between CMA and CHR, CHU in the time spent performing family planning consultation, patient medical visit, and manual vacuum aspiration (P<0.000) (Table 1).

Delivery and dressing activities are those which occupy most of the midwives' and nurses' time. However, patient care varies by health care unit, and the time used in this study is the median time spent per activity for a hospitalized patient in maternity care per day (24 hours). For example, a hospitalized patient in intensive care at CHU Yalgado Ouédraogo maternity ward receives on average six times more care time (one hour) per day than a patient hospitalized in the postpartum care unit (10 minutes). We found no significant statistical difference between the times that the different facilities take to perform this activity (p=0.801).

### Current situation in different maternity wards

Table 2 shows the number of health workers who work in the maternity wards according to their category. The maternity wards of these different facilities vary in size and, therefore, vary in terms of their staff qualifications. The CHU has the largest workforce and is the most comprehensive in terms of health worker qualifications.

**Table 2:** Breakdown of different occupational categories by maternity ward

Occupational Category	CHU	Frequency (%)	CHR	Frequency (%)	CMA	Frequency (%)
Gynecologists	11	10.9	2	4.6	5	17.9
Anesthesiologists	1	1.0	0	0	0	0
Nurse anesthetists	14	13.9	7	16.3	0	0
Surgical nurses	17	16.8	3	7.0	0	0
Midwives/Nurses	39	38.6	28	65.1	23	82.1
Orderlies	19	18.8	3	7.0	0	0

Total	101	100	43	100	28	100

Midwives and nurses are the best-represented occupational category within these facilities (see Table 3). At the CHU, they represent 38.6% of the workforce (n=39/101); at the Kaya CHR, they represent 65.1% (n=28/43); and at the CMA, they represent over 82% (n=23/28).

**Table 3:** Current situation of midwives and nurses in comparison with required staff in 2012

Occupational category			Facility	
Nurses/Midwives		CHU	CHR	CMA

				
Actual staff		39	28	23
Required staff		57	14	29
WISN Ratio		0.68	2	0.79

There are also anesthesia and surgical assistants, who work strictly as specialist nurses assisting specialist physicians. Orderlies are responsible for cleaning medical equipment and rooms.

At the CMA, specialist nurses, who are often tied up doing surgical or anaesthesia work, were not considered part of the maternity staff.

### Number of midwives and nurses required for observed activities

With respect to the retrospective data available on activities, the number of midwives and nurses required varies depending on the facility. The number of hospitalized patients in 2011 was 8752 at the CHU, 2511 at the CHR, and 4199 at the CMA.

A total of 57 nurses and midwives are required to deliver care in the CHU Yalgado Ouédraogo maternity ward. The actual number of midwives and nurses is estimated at 39. The WISN ratio of actual health workers to the required number of health workers is 0.68.

At the Kaya CHR, which is the referral centre for the Centre-Nord Region of Burkina Faso, 14 nurses and midwives are required to handle the workload. The actual number of existing staff is estimated at 28 health workers, for a WISN ratio of 2.

The Sector 30 CMA requires 29 nurses and midwives to perform all health care activities in the maternity ward. The CMA actually has 23 midwives and nurses, for an estimated WISN ratio of 0.79. This medical centre is known for performing a high number of curative and preventive activities compared to the Kaya CHR. Like the CHU, the delivery room is a very busy unit. This activity requires more time and more staff compared to other activities.

## Discussion

This study focused on midwives and nurses, though we did note that there are other occupational categories working in CHU, CHR, and CMA maternity wards. Nurses and midwives were primarily responsible for the health care activities of hospitalized patients in the different units. Unofficial staff (e.g. students, medical or health school students, and hospital interns) also assisted with curative activities. CHU deliveries are almost all done by midwives at the unit delivery room. However, in other units such as post-surgical and intensive, most medical care or resuscitations involve nurses and midwives who administer inpatient care. This task-shifting can have an effect on workload estimates in nursing and midwifery.

We also found that in the CHU delivery room, interns conduct deliveries in the course of their training and also give treatment to patients admitted to intensive care. These activities should actually be done by midwives and nurses. At the CHR and CMA levels, the distinction is less evident. It must be noted that the Yalgado Ouédraogo CHU is the training centre for medical and health students in Burkina Faso. The number of trainees could lead to underestimates of the number of official staff working in maternity services. Yet at the CHU and CMA levels, the workforce would able to cope with the workload because students were not recorded in this study.

The other occupational categories, such as physicians, frequently handle outpatient consultations and assist with other health care activities, as needed. In most of the observed activities (deliveries, caesareans and others), physicians split their time with nursing and midwife staff. Occasionally, some of the activities mentioned here were also performed by other occupational categories, but at proportionally different amounts of times. It was straightforward to determine which activities were performed by physicians and other occupational categories, but it was difficult to separate the activities performed by nurses and midwives, especially at the CHR and the CMA. It was noted that the lower on the health care pyramid, the more the entry- and mid-level health care staff perform the activities in the wards. This differs from the upper level, where specialist physicians are surrounded by every level of health care staff.

There are significant differences between the time taken to accomplish some activities at the CHU, CHR, and CMA. This is explained by the fact that some activities, such as family planning consultation and family planning counseling, are done simultaneously at the CHR and CMA, but independently, in two different rooms, at the CHU.

In other studies and WHO documents, nurses and midwives were treated jointly and even as a single entity in the estimate of health human resources [7,16,18].

Even though nurses and midwives are the most numerous among the occupational categories, there is a shortage of official staff at the CHU and CMA, unlike at the CHR, where they currently have enough staff to perform their activities. According to the results, health workers work under considerable pressure at the CHU and CMA with WISN ratios of 0.68 and 0.79, respectively. There is a tendency to say that health workers are overstaffed in big cities, but we find that these numbers are offset by facility and by demand, especially in these two major facilities (CHU Yalgado Ouedraogo and CMA 30) after this subsidy policy. In one of our previous studies, we showed that these numbers were rather concentrated at the CSPS level [11]. The workload in the maternity ward was also excessive. It is completely different at the CHR, where, with the current number of staff, health workers can generally work without pressure.

Our results from the CHU and CMA are comparable to the results found in other low-income African countries, such as Tanzania, where a severe shortage of perinatal care workers (nurses and midwives) was identified in all Dar es Salaam health care facilities [18].

Surgical and anesthesia assistants at the CMA were not considered maternity staff, as they perform activities throughout the health care centre, not solely in the maternity ward, as is the case at the CHR and CHU.

Midwives and nurses spend more time performing deliveries at the CMA than at the CHU. This is explained by the fact that at the CMA, patients arrive at the beginning of labour. They therefore require care over a longer period, unlike at the CHU, where most patients were referred by lower levels of care (CSPSs, CMAs, CHRs, or private hospitals and clinics) when they are already in labour. Furthermore, this difference is not statistically significant across facilities (p=0.080).

Burkina Faso’s policy to apply a maternal health care subsidy (SONU) is beneficial because it responds on the population’s needs. Indeed, it is desirable to also focus on the provision of care, such as hiring additional staff or simply allowing a better geographical distribution of health workers for care. However, Burkina Faso now has a good assisted delivery rate. This is a great achievement for the country. Although the country is experiencing problems in human resources, it is better placed compared to some countries in the sub-region such as Mali or Niger [7].

It is clear that the staffing shortage can have an impact on care, which could result in difficulties in attending to obstetrical complications, less time to perform tasks, compromised quality of care, and difficulty in reducing maternal mortality.

However, at the CHR, the supply of staff was much greater than the current workload. This raises the issue of geographical distribution of available human resources in health care facilities. In some African countries, this distribution was found to be very uneven between urban areas, which have a higher ratio of health care professionals, and rural areas [18-20]. The CHR in this study is in an urban area. When staff are posted to a region, they tend to stay in the region's urban area.

This study should be expanded to unofficial staff to understand their place in the workload distribution, while also incorporating all staff working in maternity (medical interns, physicians, assistants, etc.).

The strength of this study was to know the various activities carried out by nurses and midwives, and also time to complete these activities. The limitations of this study include not taking into account the services and students who are not permanent. The numbers also vary significantly over the course of a year and few health facilities were included in our study.

## Conclusion

This study showed a shortage of nurses and midwives in two health facilities in the capital of Ouagadougou after the intervention of SONU, which confirms that there is considerable demand. However, the presence of interns, which could affect the workload at the CHU, should also be taken into consideration because a similar study done in a region of Burkina showed that there was no problem in this regard. At the Kaya CHR, there is currently enough staff to handle the workload in the maternity ward, which may indicate a need to expand the analysis to other health facilities, to determine whether a redistribution of health human resources is warranted. Although Burkina Faso is focusing on the accessibility of maternal health care, it should also be focusing on the availability of human resources and, above all, their distribution across health centres to achieve MDG 4.

## List of abbreviations used

CHR: Regional hospital - Centre Hospitalier Régional; CHU: University hospital - Centre Hospitalier Universitaire; CMA: Medical Centre with Surgical Unit - Centres Médicaux avec Antennes chirurgicales; CSPS: Health centre - Centre de Santé et de Promotion Sociale; DFATD: Foreign Affairs, Trade and Development Canada; EmONC: Emergency obstetric and newborn care; GHRI: Global Health Research Initiative; HDSS: Health and Demographic Surveillance System; IDRC: International Development Research Centre; MDGs: Millennium Development Goals; MVA: Manual vacuum aspiration; SONU: Maternal health care subsidy - les soins obstétricaux et néonataux d’urgence; WISN: Workload Indicators of Staffing Need; WHO: World Health Organization.

## Competing interests

The authors declare that they have no competing interests.

## Authors' contributions

SK conceived of the idea and participated in the design of the study. AL participated in the design of the study, coordinated data collection, and conducted the analysis and writing of the manuscript. VR helped develop the idea, study design, writing, and interpretation. All authors read, commented on, and approved the manuscript.

## Declarations

The publication costs associated with this article are funded by Foreign Affairs, Trade and Development Canada and the International Development Research Centre through the Global Health esearch Initiative.

This article has been published as part of *Human Resources for Health* Volume 12 Supplement 1, 2014: Addressing the human resources for health crisis through task-shifting and retention: results from the Africa Health Systems Initiative's research component. The full contents of the supplement are available online at http://www.human-resources-health.com/supplements/12/S1
